# Muscle matters: Skeletal muscle index and body mass index impact on complications and survival in renal cancer

**DOI:** 10.1002/bco2.405

**Published:** 2024-06-13

**Authors:** Alexander Hintze Hillers, Signe Wang Bach, Atena Saito, Nessn Azawi

**Affiliations:** ^1^ Department of Urology Rigshospitalet Copenhagen Denmark; ^2^ Department of Urology Zealand University Hospital Roskilde Denmark; ^3^ Department of Urology Herlev Hospital Herlev Denmark; ^4^ Pontifical Catholic University of Campinas São Paulo Brazil; ^5^ Institute of Clinical Medicine University of Copenhagen Copenhagen Denmark

**Keywords:** body mass index (BMI), overall survival, postoperative complications, renal cell carcinoma, skeletal muscle index (SMI)

## Abstract

**Objective:**

The objective of this study is to independently assess skeletal muscle index (SMI) and body mass index (BMI) as prognostic determinants for renal cell carcinoma (RCC) and investigate their correlation with surgical outcomes.

**Patients and methods:**

A retrospective cohort study of 524 RCC patients diagnosed between August 2010 and July 2018 was conducted using data from the Zealand University Hospital Renal Cancer Database in Denmark. Patient information was extracted from electronic patient records and the National Cancer Registry and encompassed demographics, clinical factors, tumour characteristics and surgical details. SMI was calculated from a single third lumbar vertebra (L3) axial computed tomography (CT) image via CoreSlicer software and classified into high using gender‐specific thresholds. Primary outcomes focused on complications within 90 days as well as survival outcomes, and their relation with both SMI and BMI. Multivariable analysis assessed SMI's independent prognostic significance in RCC.

**Results:**

Among 524 patients, 18.5% experienced complications, with high SMI correlating significantly (*p* = 0.018) with a 72% higher complication risk. High SMI patients had a 22.7% complication rate compared to 14.5% in the low SMI group. High SMI was also linked to prolonged survival (110.95 vs. 94.87 months; *p* = 0.001), whereas BMI showed no significant survival differences (*p* = 0.326). Multivariable analysis (*n* = 522) revealed high SMI associated with improved survival (hazard ratio [HR] = 0.738; 95% CI, 0.548–0.994; *p* = 0.046). Advanced T‐stage significantly impacted mortality (T2: HR = 2.057; T3: HR = 4.361; *p* < 0.001), and each additional year of age raised mortality risk by 4.3% (HR = 1.043; *p* < 0.001).

**Conclusions:**

Higher SMI increases the risk of postoperative complications, yet it significantly improves overall survival rates. Different BMI categories lack RCC prognostic significance. The increasing incidence in RCC calls for the use of CT scan to assess SMI and aid treatment planning in patients who might benefit from preoperative interventions.

## 1 INTRODUCTION

The incidence of renal cancer has been rising steadily since the 2000s and accounted for 430 000 total cases in 2020, ranking as the 14th most common cancer worldwide.^1^ Numerous prognostic factors have already been identified, such as smoking status, hypertension, and male gender.^2^ Body mass index (BMI) is associated with increasing risk of developing renal cell carcinoma (RCC).^1^

Interestingly, data establish a correlation between higher BMI and improved cancer‐specific survival (CSS), alongside augmented recurrence‐free survival (RFS)^3^, ^4^, ^5^ and prolonged overall survival (OS) in overweight and obese patients compared to those of normal weight.^6^ Kott et al. revealed diminished postoperative complications subsequent to nephrectomy in patients with a BMI up to 30 kg/m^2^,^7^ reinforcing the ‘obesity paradox’ and emphasizing that BMI alone is an insufficient prognostic indicator for RCC outcomes. Shifting focus, the skeletal muscle index (SMI), frequently evaluated through the third lumbar vertebra (L3) index, has emerged as a promising replacement instead of BMI, accurately reflecting body composition as assessed by computed tomography (CT scan) of the L3. The threshold value for SMI was chosen based on its association with decreased survival, which is linked to weight loss exceeding 8%. This selected threshold predicts nearly half of the population's risk.^4^

Reduced SMI, signalling the presence of sarcopenia, surfaces as a risk factor for diminished OS and RFS subsequent to nephrectomy.^8^, ^9^, ^10^ Notably, a confluence of evidence suggests that patients exhibiting both sarcopenia and higher BMI tend to experience higher rates of surgical complications.^11^ Beyond the confines of RCC, SMI has demonstrated efficacy in prognosticating post‐surgical outcomes in colorectal and lung cancer, irrespective of BMI variations.^12^, ^13^

This highlights a potentially complex interplay between these measures, urging further research of the distinct roles of BMI and SMI as autonomous prognostic determinants for RCC. Thus, the present study seeks to compare BMI and SMI as independent prognostic factors for RCC and the association between both measures and surgical outcomes.

## 2 PATIENTS AND METHODS

### 2.1 Study population and data source

We conducted a retrospective cohort study, identifying 524 patients diagnosed with localized RCC from August 2010 to July 2018, using the Zealand University Hospital Renal Cancer Database. The study involved meticulous data extraction of variables including Eastern Cooperative Oncology Group (ECOG) performance status, surgical approaches, age, gender, BMI, Charlson score, pathological findings and smoking status. In cases of missing data, medical personnel involved in the patients' care reviewed patient electronic journals.

### 2.2 Body composition analysis

Body composition parameters were ascertained using a single axial CT image at the L3. Images were analysed using the free open‐source web‐based software package CoreSlicer (version 1.0.0; Montreal, Quebec, Canada),^14^ applying predefined density thresholds in Hounsfield units (HUs): 29 to +150 for skeletal muscle (SM), −190 to −30 for subcutaneous adipose tissue (SAT) and −150 to −50 for visceral adipose tissue (VAT). Total cross‐sectional areas of SM, VAT and SAT were measured in cm^2^, and average HU densities were documented for skeletal muscle density (SMD). Manual segmentation correction for the selected area was performed. Subsequently, cross‐sectional areas were height‐normalized (m^2^) to derive the SMI (cm^2^/m^2^). L3SMI was labelled high if it is more than 53 cm^2^/m^2^ for males and more than 41 cm^2^/m^2^ for females.^15^

### 2.3 Clinical data collection

Tumour attributes including clinical and post‐surgical TNM stage, Fuhrman grade and morphology, in addition to treatment details such as the type and date of surgery, were obtained from the NCR.

Preoperative details (body weight, height, smoking status, American Society of Anaesthesiologists [ASA] score), and perioperative data, encompassing complications, Clavien Grade classification, length of hospital stay (LOS), surgical blood loss refers to the bleeding that occurs during an operation and may necessitate a blood transfusion, and surgical time, were meticulously extracted from medical records by data managers from IKNL.

### 2.4 Outcome measures

The primary outcomes assessed were the impacts of SMI and BMI on oncological outcomes and complications.

## 3 RESULTS

### 3.1 Patient characteristics and outcomes

Of 524 patients stratified by the SMI, 268 had a low SMI, while 256 were classified as high SMI. The overall mortality rate in the follow‐up period was 37.4% (*n* = 197): 119 deaths occurred in the low SMI group and 78 in the high SMI group. The average age was 64.2 years (SD, 10.7 years). Patients were further categorized by BMI: 185 normal weight, 198 overweight and 141 obese. Mortality rates were 42.7% (*n* = 79) for normal weight, 35.4% (*n* = 70) for overweight and 34.0% (*n* = 48) for obese. Complications were seen in 17.8% (*n* = 33) of the normal weight, 15.2% (*n* = 30) of the overweight and 24.1% (*n* = 34) of the obese group. The correlation coefficient suggests a moderate positive relationship between BMI and SMI (*r* = 0.395, *p* < 0.001; 95% CI, 0.321 to 0.465) (Table 1).

**TABLE 1:** Basic characteristics of study population and tumour characteristics sorted in two groups according to skeletal muscle index (SMI).

		Low SMI	High SMI	*p* value
Gender	Male	180	171	0.929
	Female	88	85	
Performance score	0	152	179	0.125
	1	48	45	
	2	19	8	
	3	3	4	
	4	1	0	
	Unknown	1	0	
ASA score		145	132	0.651
	0	2	3	
	1	18	24	
	2	67	57	
	3	36	39	
	4	0	1	
T‐stage	T1	179	171	0.596
	T2	28	35	
	T3	55	46	
	T4	6	4	
Tumour types	ccRCC	26	32	0.461
	Papillary 1	18	15	
	Papillary 2	8	6	
	Chromophobe	7	2	
	Others	5	3	
	Unknown malignant	4	2	
Operation type	Open nephrectomy	14	15	0.990
	Laparoscopic nephrectomy	122	127	
	Open partial nephrectomy	8	8	
	Laparoscopic partial nephrectomy	72	80	
	Ablation of tumour	0	0	
Side of tumour	Right	110	114	0.615
	Left	119	122	
	Bilateral	0	1	
Complications	Yes	39	58	0.017*
	No	229	198	
Complication type	No complications	239	219	0.179
	Hernia	0	2	
	Fascial dehiscence	1	0	
	Reoperation	3	1	
	Other	10	14	
	Superficial infection	2	5	
	Fever	8	13	
	Renal failure	0	1	
	Intensive care unit	5	1	
Symptoms	Yes	159	163	0.197
	No	97	78	
Smoking	Never smoked	84	94	0.574
	Former smoker	69	77	
	Current smoker	69	62	
Death	Yes	119	78	<0.001*
	No	149	178	
Recurrences	Yes	48	46	0.902
	No	209	206	

Abbreviations: ASA, American Society of Anaesthesiologists; ccRCC, clear cell renal cell carcinoma.

* The observed difference is statistically significant.

### 3.2 Complications

Out of 524 patients, 97 (18.5%) had complications, with 60/524 (11.4%) postoperative complications, 35/524 (6.7%) intraoperatively and 3/524 (0.6%) being unspecified (Table 2). Sorting by BMI, 33/524 (6.3%) complications were found in patients with normal BMI, 30/524 (5.7%) complications with overweight patients and 34/524 (6.5%) with obese patients. Of these, 39/524 (7.4%) were in the low SMI group, and 58/524 (11.1%) were in the high SMI group. There was a significant association between SMI and complications (*p* = 0.018); high SMI conferred 72% increased odds of complications (95% CI, 1.099–2.693). The absolute risk for complications was 22.7% in the high SMI group compared with 14.5% in the low SMI group. However, no significant association was found between complications and BMI (*p* = 0.107) (Table 1).

**TABLE 2:** Occurrence of complications sorted in postoperative and intraoperative complications.

Complications	Frequency
Postoperative complications	
No complications	458
Fever	21
Superficial infection	7
Reoperation	4
Fascial dehiscence	1
Intensive care unit admission	6
Hernia	2
Renal failure	1
Other	24
Intraoperative complication	
Bleeding	3
Converting to open surgery	4
Converting to radical nephrectomy	9
Injury to other organs	19
No intraoperative complications	489

### 3.3 Survival analysis

**FIGURE 1:**
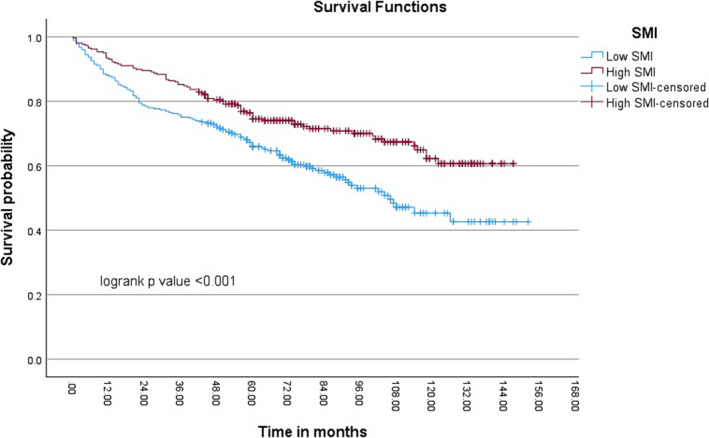
Survival time of skeletal muscle index (SMI) groups with high SMI having a bigger fraction with longer survival time.

**FIGURE 2:**
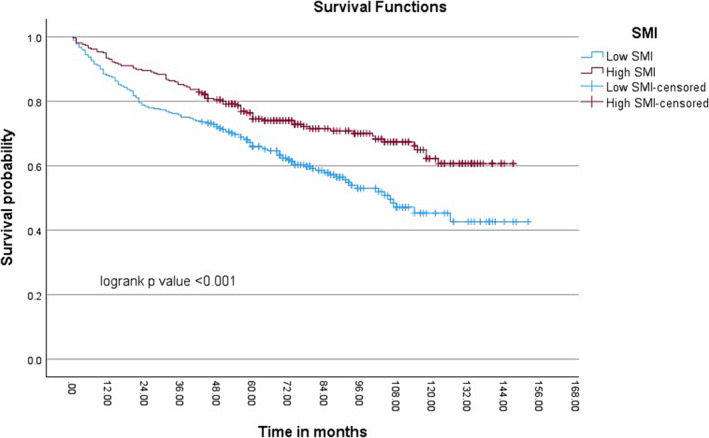
Survival time of body mass index (BMI) groups with normal BMI.

High SMI was associated with a longer mean survival of 110.95 months (95% CI, 104.28–117.62) compared to 94.87 months (95% CI, 87.29–102.45) in the low SMI group (*p* = 0.001) (see Figure 1). No significant differences in survival were noted among the BMI groups: normal BMI, 96.278 months (95% CI, 87.906–104.649); overweight BMI, 104.869 months (95% CI, 96.720–113.018); and obese BMI, 108.804 months (95% CI, 98.919–118.689) (*p* = 0.326) (Figure 2).

### 3.4 Multivariable analysis

Five hundred twenty two patients (99.6%) were included in the final multivariable analysis (Table 3). The model assessed the impact of SMI, T‐stage and age on survival. High SMI was associated with better survival (hazard ratio [HR] = 0.738; 95% CI, 0.548–0.994; *p* = 0.046). Advanced T‐stage significantly affected mortality, with T‐stage 2 and T‐stage 3 tumours having higher risks compared to T‐stage 1 (HR = 2.057; 95% CI, 1.486–2.848; *p* < 0.001 and HR = 4.361; 95% CI, 2.114–8.997; *p* < 0.001, respectively). Each additional year of age was associated with a 4.3% higher risk of mortality (HR = 1.043; 95% CI, 1.027–1.059; *p* < 0.001).

**TABLE 3:** Multiple variable analysis with cox regression. High skeletal muscle index (SMI) was associated with better survival. The higher T‐stage, the higher risk of mortality.

Multiple variable analysis with cox regression				
Variables	Hazard ratio	95% CI		*p* value
		Lower	Upper	
SMI	0.738	0.548	0.994	
T‐stage				0.046*
T‐stage (1)	1.380	0.896	2.126	<0.001*
T‐stage (2)	2.057	1.486	2.848	0.144
T‐stage (3)	4.361	2.114	8.997	<0.001*
Age	1.043	1.027	1.059	<0.001*

* The observed difference is statistically significant.

## 4 DISCUSSION

In our investigation, a substantial correlation emerged between SMI and complications, wherein a higher SMI was associated with an increased incidence of complications. Conversely, BMI exhibited no significant influence on either survival time or the rate of complications. Our multifactorial analysis identified SMI, T‐stage and age as notable prognostic factors affecting survival and mortality.

### 4.1 Survival analysis

Our study discerned a noteworthy disparity in mean survival time between patients with high and low SMIs. Our results revealed no significant variance across the three BMI groups. Notably, the obese BMI group exhibited the highest estimated mean survival time, aligning with findings from Blute et al.,^16^ who reported comparable outcomes between obese patients and those with normal or overweight BMI. Additionally, a meta‐analysis by Zhang et al.^6^ indicated that both overweight and obese individuals experienced significantly longer OS compared to normal‐weight counterparts. Awakura et al.^17^ corroborated these findings, highlighting a BMI of 23 kg/m^2^ or higher as conducive to better OS, which are consistent with our univariate and multivariate analyses among RCC patients. Furthermore, Hasselager and Gögenur^18^ in a systematic review found that sarcopenia correlated with heightened mortality rates, both short and long term, in patients undergoing major abdominal surgery. Consequently, considering higher BMI as a protective factor against mortality in RCC aligns with our results, indicating a significant positive correlation between SMI and BMI via the Pearson correlation coefficient. This correlation suggests that a higher BMI tends to be associated with a higher SMI, and both groups demonstrate improved mortality risk assessment.

Additionally, our investigation revealed that lower SMI, advanced T‐stages and an annual increase in age were associated with reduced survival times in our multifactorial analysis. The consistent recognition of sarcopenia at diagnosis as a risk factor for diminished OS in prior studies^9^, ^10^, ^19^ supports the validity of our findings. Regarding T‐stage, a majority of our cohort presented with advanced T3 stage tumours, consistent with another study demonstrating significantly higher tumour stages in sarcopenic patients, albeit in univariable analysis.^20^ Such patients might face an increased risk of recurrence, as supported by Noguchi et al.,^21^ who established a higher T‐stage as an independent predictor for poor RFS in a multivariable study. Nevertheless, further investigation within our patient cohort would be pivotal to conclusively affirm this data. Lastly, our analysis revealed that older patients experienced diminished survival times, with a 4.3% increase in the likelihood of death per year. This observation aligns with similar findings by Liu et al.^20^ and other researchers, where age was identified as an independent predictor of sarcopenia,^22^ subsequently linked to poorer OS.

### 4.2 Complications

The univariate statistical analysis demonstrated that neither the type of surgery nor gender exerted a discernible influence on the incidence of complications. However, a distinct correlation surfaced between SMI and complications. High SMI individuals displayed a notably heightened propensity for complications compared to their low SMI counterparts. This contradicts Schmeusser et al.'s prior research, which posited that sarcopenia, or low SMI, lacks predictive capacity for major postoperative complications within 90 days for individuals with non‐metastatic renal cell carcinoma (nmRCC.^23^ Nonetheless, low SMI did exhibit a correlation with reduced OS. Despite a varied distribution of complications across BMI categories highlighted in cross‐tabulation, subsequent statistical analyses failed to establish a significant correlation between BMI category and the occurrence of complications. This challenges the assertion by Maurits et al. that obesity correlates with major postoperative complications following nephrectomy.^24^ Furthermore, a previous meta‐analysis encompassing surgical outcomes post laparoscopic and partial nephrectomy in both obese and non‐obese patients revealed a higher prevalence of Clavien grade 3 or higher complications in the obese cohort.^25^ Notably, this study did not integrate observations on sarcopenia.

Regarding gender, several studies have established a link between male gender and an elevated probability of developing sarcopenia.^9^, ^10^ An examination of obese patients found that male gender independently predicted increased blood loss and longer operative durations.^26^ Intriguingly, a substantial portion of our male patients exhibited low SMI. However, it is important to note that gender did not emerge as a significant factor in either univariate or multivariate analyses in the current study.

### 4.3 Prehabilitation

Smaller studies have been done on the effect of preoperative exercise therapy on postoperative outcomes. Gillis et al.^27^ found that in patients undergoing tumour resection for colorectal cancer, a preoperative intervention had a significant positive effect on recovering to or above baseline compared to a postoperative intervention. Similarly, Valkenet et al.^28^ found that preoperative exercise intervention could reduce time of hospital stay and reduced rate of complications. Our findings may assist in further research on these topics combined in the hopes of further improving patient outcomes in both the short‐ and long‐term postoperative period.

### 4.4 Limitations of the study

This study used high‐quality cancer data to produce a better understanding of the impact both SMI and BMI have on RCC prognosis. Despite that, it is limited by the retrospective design of and by the number of the patients included, because it was conducted in a single centre. This increases the risk of selection bias within our study, and therefore, no comment can be made on whether the results are applicable for all postoperative RCC patients. However, the comparisons of country specific results are relatively reliable.

### 4.5 Conclusion

The association between higher SMI and increased postoperative complication risks, despite its significant improvement in OS rates, underscores the need to distinguish high‐risk RCC patients for precise treatment planning. However, different BMI classifications show no statistical significance in RCC prognostication, emphasizing the pivotal role of SMI assessment through CT scans.

Amidst the rising incidence of RCC, utilizing CT scans to evaluate SMI and related components emerges as a critical strategy in identifying patients poised to benefit from tailored preoperative interventions. This comprehensive approach aims not only to enhance survival but also to mitigate complications, marking a crucial advancement in RCC care planning.

In clinical decision‐making, acknowledging SMI's importance holds immense value, prompting the consideration of personalized care strategies. These findings underline the potential benefits of integrating SMI assessment into treatment paradigms, thereby bolstering survival rates and refining the care trajectory for individuals with RCC.

## CONFLICT OF INTEREST STATEMENT

The authors declare no conflicts of interest.

## AUTHOR CONTRIBUTIONS

*Conceptualization, data curation, formal analysis, methodology, writing—original draft, writing—review and editing and supervision: Nessn Azawi. Data curation and writing—review and editing: Alexander Hintze Hillers. Data curation and writing—review and editing: Signe Wang Bach. Conceptualization, writing—review and editing, data curation and methodology: Atena Saito.*

## References

[1] Bukavina L , Bensalah K , Bray F , Carlo M , Challacombe B , Karam JA , et al. Epidemiology of renal cell carcinoma: 2022 update. Eur Urol. 2022;82(5):529–542. 10.1016/j.eururo.2022.08.019 36100483

[2] Hsieh JJ , Purdue MP , Signoretti S , Swanton C , Albiges L , Schmidinger M , et al. Renal cell carcinoma. Nat Rev Dis Primers. 2017;3(1):17009. 10.1038/nrdp.2017.9 28276433 PMC5936048

[3] Bagheri M , Speakman JR , Shemirani F , Djafarian K . Renal cell carcinoma survival and body mass index: a dose‐response meta‐analysis reveals another potential paradox within a paradox. Int J Obes (Lond). 2016;40(12):1817–1822. 10.1038/ijo.2016.171 27686524

[4] Balci M , Glaser ZA , Chang SS , Herrell SD , Barocas DA , Keegan KA , et al. Differential effect of body mass index by gender on oncological outcomes in patients with renal cell carcinoma. J Cancer Res Ther. 2021;17(2):420–425. 10.4103/jcrt.JCRT_546_18 34121687

[5] Takemura K , Yonekura S , Downey LE , Evangelopoulos D , Heng DYC . Impact of body mass index on survival outcomes of patients with metastatic renal cell carcinoma in the immuno‐oncology era: a systematic review and meta‐analysis. Eur Urol Open Sci. 2022;39:62–71. 10.1016/j.euros.2022.03.002 35528786 PMC9068728

[6] Zhang J , Chen Q , Li ZM , Xu XD , Song AF , Wang LS . Association of body mass index with mortality and postoperative survival in renal cell cancer patients, a meta‐analysis. Oncotarget. 2018;9(17):13959–13970. 10.18632/oncotarget.24210 29568408 PMC5862629

[7] Kott O , Golijanin B , Pereira JF , Chambers A , Knasin A , Tucci C , et al. The BMI paradox and robotic assisted partial nephrectomy. Front Surg. 2020;6:74. 10.3389/fsurg.2019.00074 31998743 PMC6962129

[8] Higgins MI , Martini DJ , Patil DH , Nabavizadeh R , Steele S , Williams M , et al. Sarcopenia and modified Glasgow Prognostic Score predict postsurgical outcomes in localized renal cell carcinoma. Cancer. 2021;127(12):1974–1983. 10.1002/cncr.33462 33760232

[9] Lee J , Suh J , Song C , You D , Jeong IG , Hong B , et al. Association between sarcopenia and survival of patients with organ‐confined renal cell carcinoma after radical nephrectomy. Ann Surg Oncol. 2022;29(4):2473–2479. 10.1245/s10434-021-10881-7 34625877

[10] Psutka SP , Boorjian SA , Moynagh MR , Schmit GD , Costello BA , Thompson RH , et al. Decreased skeletal muscle mass is associated with an increased risk of mortality after radical nephrectomy for localized renal cell cancer. J Urol. 2016;195(2):270–276. 10.1016/j.juro.2015.08.072 26292038

[11] Peyton CC , Heavner MG , Rague JT , Krane LS , Hemal AK . Does sarcopenia impact complications and overall survival in patients undergoing radical nephrectomy for stage III and IV kidney cancer? J Endourol. 2016;30(2):229–236. 10.1089/end.2015.0492 26418428

[12] Boer BC , De Graaff F , Brusse‐Keizer M , Bouman DE , Slump CH , Slee‐Valentijn M , et al. Skeletal muscle mass and quality as risk factors for postoperative outcome after open colon resection for cancer. Int J Colorectal Dis. 2016;31(6):1117–1124. 10.1007/s00384-016-2538-1 26876070

[13] Martin L , Birdsell L , MacDonald N , Reiman T , Clandinin MT , McCargar LJ , et al. Cancer cachexia in the age of obesity: skeletal muscle depletion is a powerful prognostic factor, independent of body mass index. J Clin Oncol. 2013;31(12):1539–1547. 10.1200/JCO.2012.45.2722 23530101

[14] Mullie L , Afilalo J . CoreSlicer: a web toolkit for analytic morphomics. BMC Med Imaging. 2019;19(1):15. 10.1186/s12880-019-0316-6 30744586 PMC6371488

[15] Portal D , Hofstetter L , Eshed I , Dan‐Lantsman C , Sella T , Urban D , et al. L3 skeletal muscle index (L3SMI) is a surrogate marker of sarcopenia and frailty in non‐small cell lung cancer patients. Cancer Manag Res. 2019;11:2579–2588. 10.2147/CMAR.S195869 31114324 PMC6497853

[16] Blute ML , Zorn K , Grimes M , Shi F , Downs TM , Jarrard DF , et al. Extreme obesity does not predict poor cancer outcomes after surgery for renal cell cancer. BJU Int. 2016;118(3):399–407. 10.1111/bju.13381 26589741 PMC6996861

[17] Awakura Y , Nakamura E , Ito N , Yamasaki T , Kamba T , Kamoto T , et al. Influence of body mass index on prognosis of Japanese patients with renal cell carcinoma. Urology. 2007;70(1):50–54. 10.1016/j.urology.2007.03.034 17656207

[18] Hasselager R , Gögenur I . Core muscle size assessed by perioperative abdominal CT scan is related to mortality, postoperative complications, and hospitalization after major abdominal surgery: a systematic review. Langenbecks Arch Surg. 2014;399(3):287–295. 10.1007/s00423-014-1174-x 24535479

[19] Yuxuan L , Junchao L , Wenya L . The role of sarcopenia in treatment‐related outcomes in patients with renal cell carcinoma: a systematic review and meta‐analysis. Medicine (Baltimore). 2022;101(43):e31332. 10.1097/MD.0000000000031332 36316941 PMC9622586

[20] Liu Q , Yang J , Chen X , Yang J , Zhao X , Huang Y , et al. Prognostic significance of sarcopenia and systemic inflammation for patients with renal cell carcinoma following nephrectomy. Front Oncol. 2022;12:1047515. 10.3389/fonc.2022.1047515 36591466 PMC9798277

[21] Noguchi G , Kawahara T , Kobayashi K , Tsutsumi S , Ohtake S , Osaka K , et al. A lower psoas muscle volume was associated with a higher rate of recurrence in male clear cell renal cell carcinoma. PLoS ONE. 2020;15(1):e0226581. 10.1371/journal.pone.0226581 31895931 PMC6939903

[22] Khan AI , Psutka SP , Patil DH , Hong G , Williams MA , Bilen MA , et al. Sarcopenia and systemic inflammation are associated with decreased survival after cytoreductive nephrectomy for metastatic renal cell carcinoma. Cancer. 2022;128(11):2073–2084. 10.1002/cncr.34174 35285950

[23] Schmeusser BN , Midenberg E , Palacios AR , Ali AA , Patil DH , Higgins M , et al. Low skeletal muscle as a risk factor for worse survival in nonmetastatic renal cell carcinoma with venous tumor thrombus. Clin Genitourin Cancer. 2023;21(4):475–482.e4. 10.1016/j.clgc.2023.04.005 37210313

[24] Maurits JSF , Sedelaar JPM , Aben KKH , Kiemeney LALM , Vrieling A . The association of body composition with postoperative complications and length of hospital stay after radical or partial nephrectomy in patients with renal cell cancer: a multicenter population‐based cohort study. Transl Androl Urol. 2022;11(12):1667–1679. 10.21037/tau-22-367 36632160 PMC9827407

[25] Aboumarzouk OM , Stein RJ , Haber GP , Kaouk J , Chlosta PL , Somani BK . Laparoscopic partial nephrectomy in obese patients: a systematic review and meta‐analysis. BJU Int. 2012;110(9):1244–1250. 10.1111/j.1464-410X.2012.11094.x 22471614

[26] Abdullah N , Dalela D , Barod R , Larson J , Johnson M , Mass A , et al. Robotic partial nephrectomy for renal tumours in obese patients: perioperative outcomes in a multi‐institutional analysis. Can Urol Assoc J. 2015;9(11–12):E859–E862. 10.5489/cuaj.3197 26788235 PMC4707905

[27] Gillis C , Li C , Lee L , Awasthi R , Augustin B , Gamsa A , et al. Prehabilitation versus rehabilitation: a randomized control trial in patients undergoing colorectal resection for cancer. Anesthesiology. 2014;121(5):937–947. 10.1097/ALN.0000000000000393 25076007

[28] Valkenet K , van de Port IGL , Dronkers JJ , de Vries WR , Lindeman E , Backx FJG . The effects of preoperative exercise therapy on postoperative outcome: a systematic review. Clin Rehabil. 2011;25(2):99–111. 10.1177/0269215510380830 21059667

